# *Salmonella* Saintpaul outbreak associated with cantaloupe consumption, the United Kingdom and Portugal, September to November 2023

**DOI:** 10.1017/S0950268824000670

**Published:** 2024-05-06

**Authors:** Luke J. McGeoch, Ann Hoban, Clare Sawyer, Hussein Rabie, Incident Team, Anaïs Painset, Lynda Browning, Derek Brown, Caitlin McCarthy, Andrew Nelson, Ana Firme, Ângela Pista, Joana Moreno, João Vieira Martins, Leonor Silveira, Jorge Machado, Paula Vasconcelos, Oluwakemi Olufon, Carmellie Inzoungou-Massanga, Amy Douglas, Jacquelyn McCormick, Lesley Larkin, Sooria Balasegaram

**Affiliations:** 1 Field Service South-East and London, Health Protection Operations Division, UK Health Security Agency, London, UK; 2 Field Epidemiology Training Programme, UK Health Security Agency, London, UK; 3 Gastrointestinal Infections and Food Safety (One Health) Division, Clinical and Public Health Group, UK Health Security Agency, London, UK; 4 UK Health Security Agency, London, UK; 5 Food Standards Agency, London, UK; 6 Gastrointestinal Bacteria Reference Unit, Clinical and Public Health Group, UK Health Security Agency, London, UK; 7 Clinical and Protecting Health Directorate, Public Health Scotland, Glasgow, UK; 8 Scottish Microbiology Reference Laboratory (SMiRL), Glasgow, UK; 9 Communicable Disease Surveillance Centre, Public Health Wales, Cardiff, UK; 10 Public Health Emergencies Operations Centre, Directorate-General of Health, Lisbon, Portugal; 11 National Reference Laboratory for Gastrointestinal Infections, Department of Infectious Diseases, National Institute of Health Doutor Ricardo Jorge, Lisbon, Portugal; 12 Directorate of Information and Analysis, Directorate-General of Health, Lisbon, Portugal; 13 Rapid Investigation Team, Field Services, Health Protection Operations Division, UK Health Security Agency, London, UK

**Keywords:** cantaloupe, melon, outbreak, *Salmonella*, *Salmonella* Saintpaul

## Abstract

In September 2023, the UK Health Security Agency identified cases of *Salmonella* Saintpaul distributed across England, Scotland, and Wales, all with very low genetic diversity. Additional cases were identified in Portugal following an alert raised by the United Kingdom. Ninety-eight cases with a similar genetic sequence were identified, 93 in the United Kingdom and 5 in Portugal, of which 46% were aged under 10 years. Cases formed a phylogenetic cluster with a maximum distance of six single nucleotide polymorphisms (SNPs) and average of less than one SNP between isolates. An outbreak investigation was undertaken, including a case–control study. Among the 25 UK cases included in this study, 13 reported blood in stool and 5 were hospitalized. One hundred controls were recruited via a market research panel using frequency matching for age. Multivariable logistic regression analysis of food exposures in cases and controls identified a strong association with cantaloupe consumption (adjusted odds ratio: 14.22; 95% confidence interval: 2.83–71.43; *p*-value: 0.001). This outbreak, together with other recent national and international incidents, points to an increase in identifications of large outbreaks of *Salmonella* linked to melon consumption. We recommend detailed questioning and triangulation of information sources to delineate consumption of specific fruit varieties during *Salmonella* outbreaks.

## Key results

- Between September and November 2023, an outbreak of *Salmonella* Saintpaul occurred in the United Kingdom and Portugal, with 98 confirmed cases identified.
- On phylogeny, a monophyletic branch contained all cases, with low genetic diversity, suggestive of a common source.
- A case–control study identified a strong association between case status and cantaloupe consumption (adjusted odds ratio: 14.22; 95% confidence interval: 2.83–71.43).
- There has been an increase in outbreaks of *Salmonella* linked to cantaloupe and other melon varieties.
- Detailed case questioning and triangulation of information sources are needed to delineate fresh produce exposures, particularly for children.

*Salmonella* comprises more than 2 600 distinct serovars of gram-negative bacteria, with over half belonging to *Salmonella enterica* subsp. *enterica*, many of which can infect and cause disease in humans and are spread via contaminated food and person-to-person transmission [1]. They typically cause fever, abdominal pain, diarrhoea, nausea, and vomiting, with some people experiencing more severe and even life-threatening illness that requires hospitalization. In recent years, there have been several large national or international outbreaks of *Salmonella* linked to consumption of melon [2–5]. In the present report, we describe an outbreak of *Salmonella* Saintpaul affecting 98 people in the United Kingdom and Portugal found to be associated with consumption of cantaloupe.

An outbreak of *Salmonella* Saintpaul infections, with isolates for all cases falling within a 5-single nucleotide polymorphism (SNP) single linkage cluster based on whole genome sequencing (WGS), was identified by the UK Health Security Agency (UKHSA). Further cases in Portugal and multiple other countries were identified using the European Centre of Disease Prevention and Control’s EpiPulse platform [6]. We established an incident management team on 26 October 2023 including public health colleagues from UKHSA, Public Health Scotland, and Public Health Wales, and representatives from the UK Food Standards Agencies (FSA England and FSA Wales) and Food Standards Scotland. A confirmed case was defined as a person with an isolate testing positive for *Salmonella* Saintpaul within the 5-SNP single linkage cluster with UKHSA SNP designation 1.497.576.672.746.812% [7], with a sample receipt date (the date that samples were received by the relevant national reference laboratory) on or after 1 September 2023. An alternative case definition for laboratories using the Enterobase Hierarchical cgMLST clustering included cases falling within the HC5_380529 cluster, or for laboratories using the SeqSphere cgMLST scheme, Complex Type (CT) 20311. Information on this UK incident was disseminated internationally via EpiPulse on 27 October 2023.

**Figure 1.**
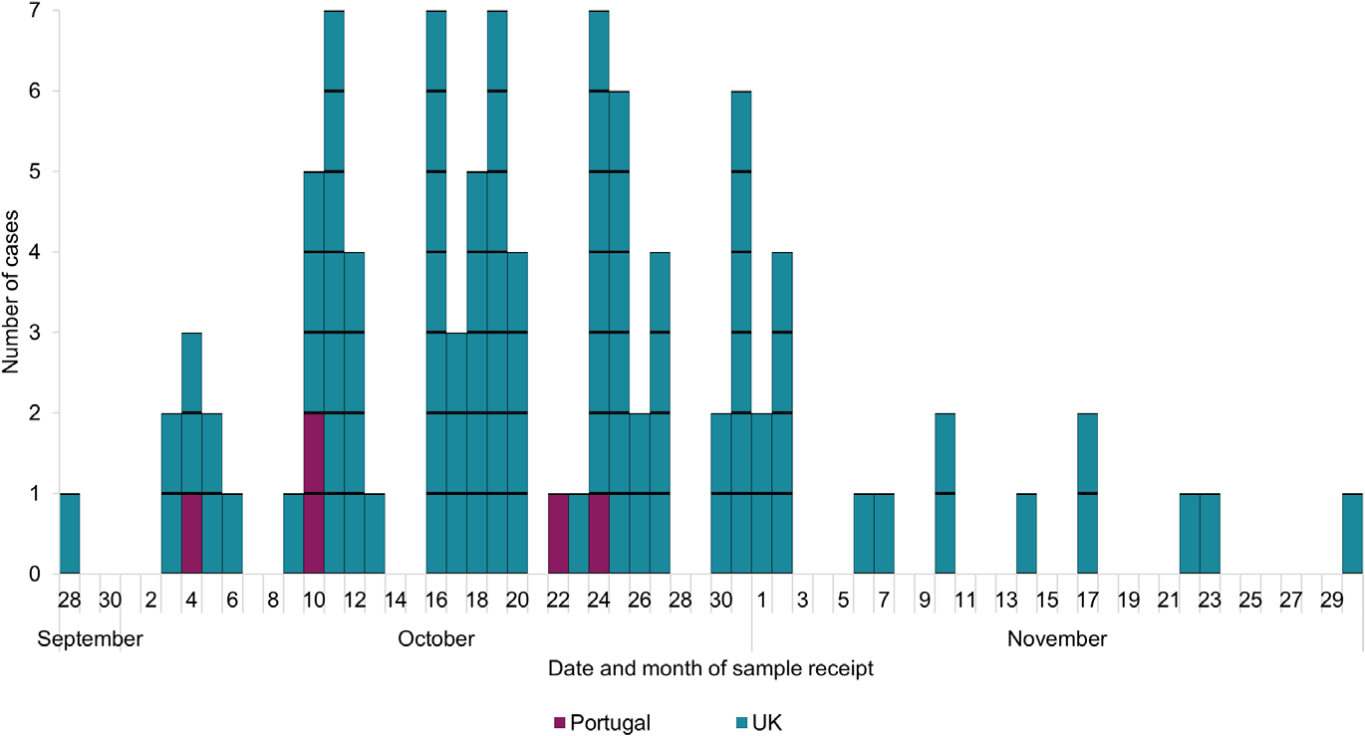
Epidemic curve for confirmed cases of *Salmonella* Saintpaul (*n* = 98), the United Kingdom and Portugal, September to November 2023.

There were 93 confirmed cases in the United Kingdom, geographically dispersed in England (*n* = 78), Scotland (*n* = 10), and Wales (*n* = 5), with sample receipt dates from 28 September 2023 to 30 November 2023 (Figure 1). Sample receipt date is used as information on onset of symptoms was not available for all cases. Cases had a median age of 20 years (range 10 months to 89 years); 28% were aged under 5 years and 43% under 10 years. The majority (63%) were female. Five cases were identified in Portugal, with sample dates from 4 October 2023 to 24 October 2023. These cases had a median age of 3 years (range 2–8 years), and 80% were female. Following the UK alert, proactive identification of *Salmonella* cases and additional whole genome sequencing were performed.

Faecal samples testing positive for *Salmonella* in all diagnostic laboratories in Great Britain are routinely sent to the UKHSA Gastrointestinal Bacteria Reference Unit (England and Wales) or Scottish Microbiology Reference Laboratories (Scotland) for characterization. The outbreak cases formed a cluster with a maximum distance of six SNPs and average of less than one SNP between isolates (Supplementary Figure S1; the Supplementary Material is available on the Cambridge Core website). The phylogeny represents a monophyletic branch containing all cases with low genetic diversity, suggestive of a common source. A representative sequence is available in the Short Read Archive for comparison, accession number SRR26450426. A representative sequence for the Portugal cases provided by the National Reference Laboratory for Gastrointestinal Infections in Portugal was found to be within the same 0-SNP cluster as the majority of UK sequences, with UKHSA SNP designation 1.497.576.672.746.812.949.

Exploratory interviews using an open-ended, anthropological approach were undertaken for five cases in England and Wales for hypothesis generation. The hypothesis that a fresh produce item was the vehicle of transmission was then investigated using a case–control study. Twenty-five UK cases, none of whom had previously been interviewed, completed a trawling questionnaire focusing on fresh produce and egg consumption in the 7 days preceding symptom onset. All were primary cases present in the United Kingdom during this 7-day period. Symptom onset dates ranged from 20 September 2023 to 1 November 2023 (Supplementary Figure S2; the Supplementary Material is available on the Cambridge Core website). Information on food exposures was not collected for Portuguese cases, so these cases were not included in the case–control study. One hundred controls were recruited via a market research panel company and frequency matched by age group to cases, given the high proportion of cases aged under 10 years. For cases and controls in this age group, parents completed questionnaires. A higher percentage of cases (48%) were aged under 10 years compared with controls (38%). Fifty-six per cent of cases were female, compared to 54% of controls. Twenty-four (96%) cases reported diarrhoea, 13 (52%) reported blood in stool, and five (20%) reported being admitted to hospital. Notably, melon consumption was reported by 13 (52%) cases, compared with 24 (24%) controls.

We computed odds ratios (OR), 95% confidence intervals (95% CI), and *p*-values using Pearson’s chi-squared or Fisher’s exact test. Exposures present in ≥20% cases with an OR >1.00 and *p*-value <0.20 were considered for inclusion in a multivariable model using logistic regression with a forward stepwise approach. Age group was included a priori given incomplete frequency matching. In univariable analyses, being a case was associated with consumption of cantaloupe (OR 12.57, 95% CI 3.20–65.21) and strawberries (3.62, 1.32–9.94) (Table 1). There was no association with age, sex, consumption of other melon varieties (galia, honeydew, watermelon, ‘other’), or place of purchase. In the multivariable analysis, being a case was again associated with consumption of cantaloupe (14.22, 2.83–71.43) and strawberries (4.59, 1.38–15.25). In sensitivity analyses conducted to investigate possible under-ascertainment of cantaloupe consumption, being a case was strongly associated with a composite variable combining consumption of cantaloupe and ‘other’ unspecified melon varieties (13.91, 3.21–60.21), weakly associated with consumption of any type of melon (2.83, 1.09–7.36) and not associated with consumption of yellow (honeydew, galia, ‘yellow’) melon varieties (2.28, 0.78–6.64).

**Table 1.** Results of multivariable analysis for case–control study including confirmed cases of *Salmonella* Saintpaul (*n* = 25) and controls (*n* = 100)

Exposure	Cases		Controls		aOR	95% CI	*p*-value
	*n*	%	*n*	%			
*Food exposures*							
Cantaloupe	7	28	3	3	14.22	2.83–71.43	0.001
Strawberries	14	56	26	26	4.59	1.38–15.25	0.013
*Age group*							
0–4 years	7	28	19	19	Ref	Ref	Ref
5–9 years	5	20	19	19	0.93	0.21–4.14	0.923
18–59 years	6	24	30	30	1.09	0.24–4.90	0.911
≥60 years	7	28	32	32	2.00	0.42–9.50	0.382

*Note*: A single case in the 10–17 years age group in the case–control study was included in the 18–59 years age group category in analyses. aOR, adjusted odds ratio; CI, confidence interval.

Our wider epidemiological investigations in the United Kingdom, including contact with cases and settings that were not included in the case–control study, provided additional evidence for a link with cantaloupe consumption. First, in hypothesis-generating exploratory interviews, all five cases confirmed melon consumption. Three of these cases reported consuming cantaloupe, one may have consumed cantaloupe, and one could not recall the variety consumed. Second, three educational settings were identified, that were each attended by multiple (up to three) cases. Catering or facilities managers were contacted for each of these settings and asked to provide information, including meal cards, which provide details of food items served to children at the setting during the relevant time period of the outbreak. These settings all served melon in the week preceding symptom onset of at least one case; two settings confirmed that they had served cantaloupe and the variety of melon served in the third setting was unknown. However, it was not possible to ascertain individual food items consumed by cases attending these educational settings. Third, cases who completed a targeted questionnaire were asked to provide consent and details to permit purchasing information from supermarket loyalty cards to be accessed by the Food Standards Agency. Ten cases provided supermarket loyalty card details, all for the same supermarket. Three of these cases had reported cantaloupe consumption in targeted questionnaires. Purchasing information was obtained for 7 of these 10 cases and revealed that all had purchased cantaloupe prior to symptom onset; three cases had also purchased honeydew melon and one case had purchased galia melon. While it is not possible to confirm that these cases actually consumed cantaloupe, these data suggest underreporting of this exposure.

Given the lead time to complete serotyping and whole genome sequencing of faecal isolates from cases, the time taken to identify cantaloupe as the suspected vehicle of transmission, and the relatively short shelf-life of melons and other fresh produce items, it was not possible to obtain contemporaneous samples of cantaloupe for microbiological testing. Furthermore, due to these factors and the rapid offset of the outbreak, specific public health control measures (such as product recall) were not instigated. Food traceback investigations regarding the source of the produce are ongoing.

In this report, we have described an outbreak of *Salmonella* Saintpaul in the United Kingdom and Portugal associated with reported consumption of cantaloupe. Almost half of cases were children aged under 10 years. A case–control study was conducted using UK cases and frequency-matched controls recruited via a market research panel. In the United Kingdom, supply chains for fresh fruit show seasonal variation, which may account for the sharp rise and tail of the outbreak. The European Food Safety Authority has identified a range of factors that increase the risk of contamination of melons with *Salmonella*, including contact with and proximity of agricultural production and processing systems to animal reservoirs, use of contaminated water in agricultural production, and contamination or cross-contamination during or after harvest [8]. *Salmonella* outbreaks previously linked to melon in recent years include a multi-European country *Salmonella* Braenderup outbreak in 2021 linked to consumption of imported galia melons [2], and a 2023 outbreak of *Salmonella* Sundsvall and *Salmonella* Oranienburg with high severity in the United States and Canada linked to cantaloupe-containing products [3]. As in this outbreak, these outbreaks were characterized by a predominance of cases in young children and older adults, who are more susceptible to severe disease. Cantaloupe consumption has been linked with outbreaks of other *S. enterica* subsp. *enterica* serovars (including *Salmonella* Saintpaul), *Campylobacter jejuni*, *Escherichia coli* O157:H7, *Listeria monocytogenes*, and norovirus [4, 5, 9]. Previous outbreaks of *Salmonella* Saintpaul have been linked with consumption of ground beef (US, 2023), cucumber (US, 2013), alfalfa sprouts (US, 2009), jalapeño and serrano peppers (US, 2008), cantaloupe (Australia, 2006), unpasteurized orange juice (US, 2005), mango (US, 2001), and beansprouts (UK, 1988) [3, 5, 10–12].

Our findings are subject to several limitations. First, a minority of cases reported cantaloupe consumption. Data from supermarket loyalty cards suggested underreporting of the exposure, and parents may have been unaware of consumption by children in nursery and primary school settings. Difficulty ascertaining consumption of melon varieties was also encountered in the 2021 *Salmonella* Braenderup outbreak [2]. However, grouping different varieties of melon within sensitivity analyses did not provide an alternative explanation. Cross-contamination of other food products is also possible. Second, while recruitment of controls using a market research panel permitted frequency matching and a timely case–control study, controls may not be wholly representative of the general population. Third, the time requirement for sample processing and whole genome sequencing delayed case questionnaires, leading to challenges with memory recall, delayed food traceback, and preventing contemporaneous microbiological testing of food samples.

In conclusion, we describe an outbreak of *Salmonella* Saintpaul with cases distributed throughout the United Kingdom and in Portugal. Epidemiological analysis provides evidence for a link with cantaloupe. In light of this and other recent large outbreaks of *Salmonella* linked to melon consumption, cantaloupe and other melon varieties should be considered as potential sources of infection during future *Salmonella* outbreaks. Furthermore, given the potential for under-ascertainment of consumption of specific varieties of fruit and vegetable products during outbreak investigations, particularly when a high proportion of cases attend educational or childcare settings, detailed questioning is needed to delineate specific fresh produce exposures, together with triangulation with data from other information sources.

## Supporting information

McGeoch et al. supplementary material McGeoch et al. supplementary material

## Data Availability

The data used in this investigation contain personal identifiable information. Anonymized information required to reproduce these results is available from the corresponding author on reasonable request.

## Supplementary material

For supplementary material accompanying this paper visit http://doi.org/10.1017/S0950268824000670. click here to view supplementary material

## Investigators

Adam Crewdson, Agnieszka Bird, Ana Firme, Anita Shah, Carol Stocker, Carrie Pollock, Catriona Angel, Christopher Williams, Claire Brown, Deepti Kumar, Donna Haskins, Ellen Heinsbroek, Emma O’Brien, Gauri Godbole, Gemma Lyness, Heather Charles, Jacob Hargreaves, Janine Thoulass, Joanna Brown, Johanna Jackson, Joseph Hibbert, Kay Ratcliffe, Lewis Peake, Luke Guinness, Marie Chattaway, Mark Tissington, Matilda Jackson-Hamilton, Matthew Neilson, Megan Bardsley, Neethu Kunnanath, Nick Young, Paul Millar, Rob Johnston, Satheesh Nair, Skye Firminger, Tina Potter, Toyin Ejidokun, Trish Mannes, and Vanessa Wong.
