# Supplementary material for: Salmonella Saintpaul outbreak associated with cantaloupe consumption, the United Kingdom and Portugal, September to November 2023
Source: Epidemiol Infect. 2024 May 6;152:e78. doi: 10.1017/S0950268824000670 (PMC11106726; doi:10.1017/S0950268824000670)

***Epidemiology and Infection***

***Salmonella* Saintpaul outbreak associated with cantaloupe consumption, United Kingdom and Portugal, September 2023 to November 2023**

Luke J. McGeoch, Ann Hoban, Clare Sawyer, Hussein Rabie, Incident Team, Anaïs Painset, Lynda Browning, Derek Brown, Caitlin McCarthy, Andrew Nelson, Ana Firme, Ângela Pista, Joana Moreno, João Vieira Martins, Leonor Silveira, Jorge Machado, Paula Vasconcelos, Oluwakemi Olufon, Carmellie Inzoungou-Massanga, Amy Douglas, Jacquelyn McCormick, Lesley Larkin, Sooria Balasegaram

**Supplementary Material**

**Supplementary Figure S1: Phylogenetic tree of *Salmonella* Saintpaul outbreak sequences (n=91), England, Scotland and Wales, September – November 2023**


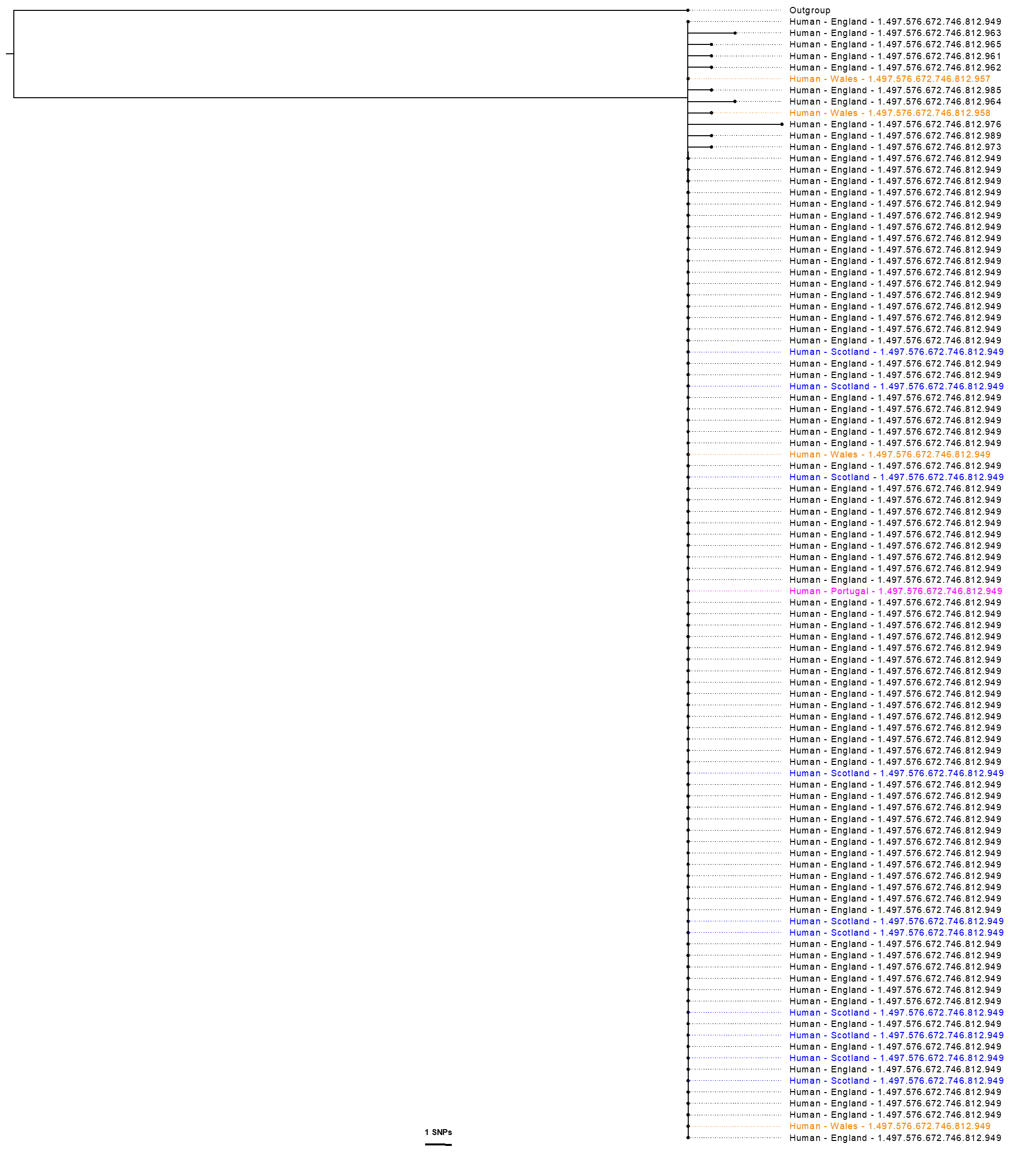


Faecal samples testing positive for *Salmonella* in all diagnostic laboratories in Great Britain are routinely sent to the UKHSA Gastrointestinal Bacteria Reference Unit (England and Wales) or Scottish Microbiology Reference Laboratories (Scotland) for characterisation, including whole genome sequencing. The outbreak cases formed a cluster with a maximum distance of six SNPs and average of less than one SNP between isolates. The phylogeny represents a monophyletic branch containing all cases with low genetic diversity, suggestive of a common source. A representative sequence is available in the Short Read Archive for comparison, accession number SRR26450426. A representative sequence for the Portugal cases provided by the National Reference Laboratory for Gastrointestinal Infections in Portugal was found to be within the same 0-SNP cluster as the majority of UK sequences, with UKHSA SNP designation 1.497.576.672.746.812.949.

**Supplementary Figure S2: Epidemic curve by symptom onset date for confirmed UK cases of *Salmonella* Saintpaul who completed a targeted questionnaire (n=26), September – November 2023**


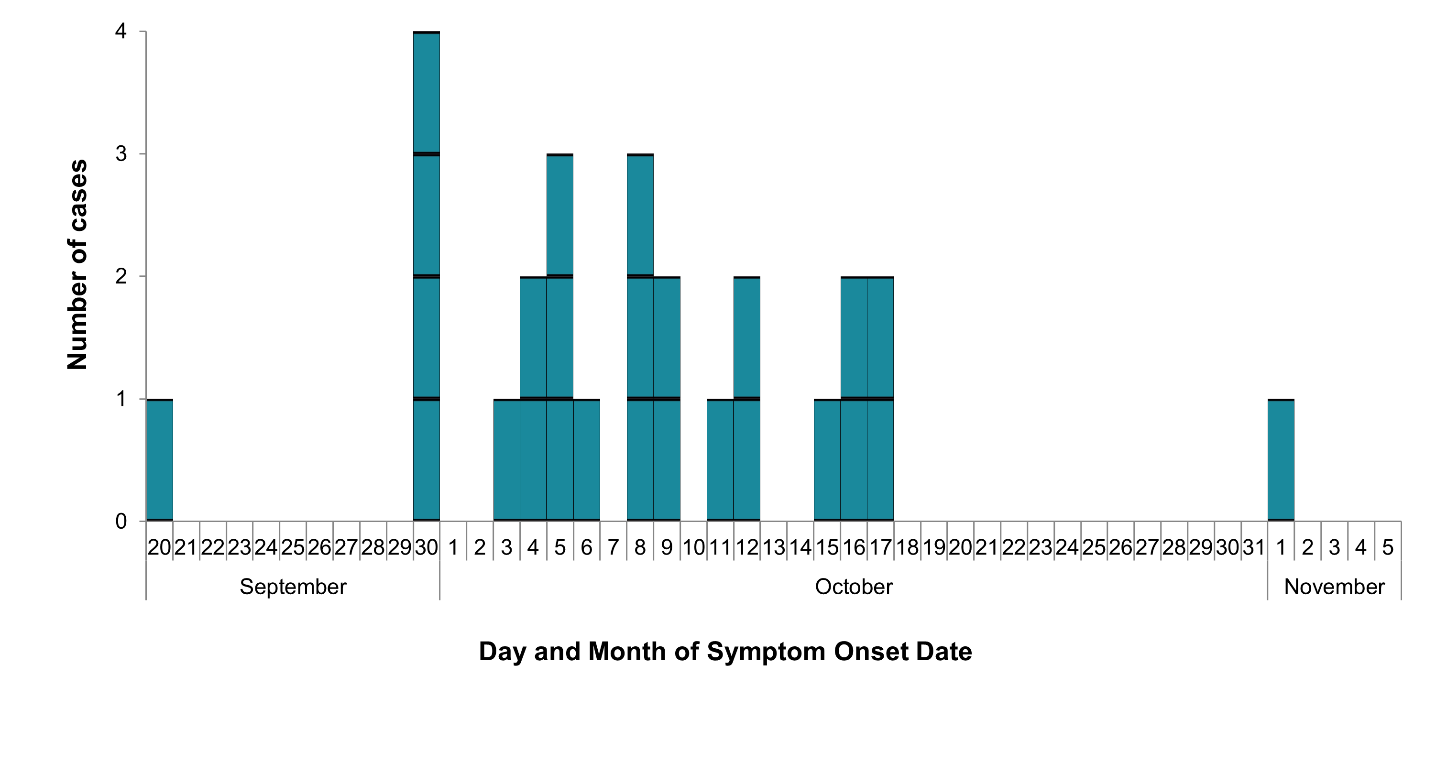

Supplement: McGeoch et al. supplementary material [file S0950268824000670sup001.docx]
